# Development of a remote implementation support strategy to enhance integration of depression treatment into primary care settings in rural India

**DOI:** 10.3389/fpubh.2024.1439997

**Published:** 2024-12-06

**Authors:** Gloria A. Pedersen, Juliana Restivo Haney, Abhishek Singh, Shivangi Choubey, Ameya Bondre, Vorapat Vorapanya, Anant Bhan, Deepak Tugnawat, Vikram Patel, John A. Naslund, Rohit Ramaswamy

**Affiliations:** ^1^Mental Health for All Lab, Department of Global Health and Social Medicine, Harvard Medical School, Boston, MA, United States; ^2^Department of Psychology, West Virginia University, Morgantown, WV, United States; ^3^Bhopal Hub, Sangath, Bhopal, Madhya Pradesh, India; ^4^Gillings School of Global Public Health, University of North Carolina at Chapel Hill, Chapel Hill, NC, United States; ^5^Department of Global Health and Population, Harvard T.H. Chan School of Public Health, Boston, MA, United States; ^6^James M. Anderson Center for Health Systems Excellence, Cincinnati Children’s Hospital Medical Center, Cincinnati, OH, United States

**Keywords:** depression, mental health, global health, implementation science, primary care

## Abstract

**Introduction:**

Contextually responsive implementation support strategies are needed to enhance the integration of mental health services into primary health care. Technical assistance is widely used as a core “capacity building” strategy, primarily for increasing the motivation and capacity of individuals (e.g., frontline workers) to adopt evidence-based interventions in healthcare settings. This article documents the systematic design of a technical assistance strategy for supporting primary care staff (e.g., nurses) in integrating depression care, from screening to treatment, aligned with a non-communicable disease program across seven health facilities in the Sehore District of Madhya Pradesh, India.

**Methods:**

We conducted a mapping exercise of local health facilities with dedicated staff and a literature review to inform the development of the technical assistance strategy.

**Results:**

Reporting guidelines guided the structure of the strategy protocol. The evidence-supported strategy, called Remote Coaching Support, is tailored to the local setting. It uses quality improvement methods like the Plan-Do-Act-Study cycle and training materials to help coaches deliver support remotely via video/phone-based calls and WhatsApp messaging with the overall goal of increasing uptake and fidelity of depression screening and referral among primary care staff in alignment with an existing non-communicable diseases program.

**Conclusion:**

The development of Remote Coaching Support involved iterative improvements through team meetings and practice-training feedback, though limitations exist due to a lack of systematic implementation standards, especially in this setting. This strategy will be tested in increasing scales to refine its application, with effectiveness and acceptability results to be evaluated in a randomized control trial.

## 1 Introduction

Depression affects over 50 million people in India, yet almost 80% of those affected do not have access to care (1, 2). Although various research studies have shown that clinical interventions for depression, delivered in primary care, are effective and cost-effective globally, including in India, evidence on models that promote sustainable implementation of these interventions in primary care is lacking (3). Integration of depression care at the primary care level is promoted globally as a feasible strategy for addressing the mental health treatment gap, with the largest body of research supporting a multi-component collaborative care model (4–6). This model typically involves a team of frontline health workers with different levels of training offering evidence-based pharmacological and non-pharmacological treatments in a stepped-care approach and ensuring coordinated care management through continuous symptom monitoring and patient-centered interventions (4).

However, these models are complex and must contextually reflect real-world practices and priorities to be successful: A recent rapid review found that although 14 effective collaborative mental health care models across nine different LMICs shared “successful ingredients,” specifics of the models and how they were implemented were unique to each setting, highlighting the significance of tailoring the model to the local context (4). Relatedly, there remain significant challenges to supporting the uptake, delivery, and sustainability of these mental health interventions within routine care, including insufficient staff training, low motivation and variable competencies of providers, heavy workload for frontline health workers, and negative attitudes about mental health conditions (7, 8). In India, these concerns are further complicated because mental health care remains significantly under-resourced, characterized by a shortage of specialists, insufficient financial allocation due to competing health program priorities, and existing health system challenges impeding routine service delivery (9).

In 2011, a team leading the Program for Improving Mental Health Care (PRIME) project in Madhya Pradesh, India partnered with the local Department of Health Services, Government of Madhya Pradesh and other key stakeholders to design and evaluate a comprehensive mental health care plan and understand the facilitators and barriers for integrating mental health interventions into primary care systems (9, 10). The research identified that employing dedicated staff (e.g., nurses) and ensuring a dedicated workspace was key to successfully delivering stepped depression care in government-based health facilities. To address barriers to sustaining uptake of the model, key takeaways from PRIME included the need to map out existing health facility procedures and to explore specific implementation support strategies, like a technical assistance team, to help overcome challenges and enhance the integration of depression care delivery (9, 10).

The field of implementation science focuses on developing, testing, and utilizing a range of implementation strategies to support the systematic uptake of evidence-based interventions, like collaborative care models, into routine care (11, 12). A classification system has been developed that groups these strategies into five distinct categories (dissemination, implementation process, integration, capacity-building, and scale-up) according to who will enact them and what action(s) the strategy targets (13).

In response, the ESSENCE program was launched (14) with the overarching goal of scaling up evidence-based depression care in rural communities in Madhya Pradesh, India. A key component of ESSENCE involved a cluster-randomized controlled trial, referred to as the “Implementation Trial.” This trial aimed to design and evaluate an implementation support strategy to help primary care facility staff adopt a collaborative care package for depression, based on the WHO Mental Health Gap Action Program (mhGAP) (15). The implementation strategy is intended to be in alignment with the Indian government’s rollout of the Ayushman Bharat initiative, which aims to expand the scope of primary care to include the management of non-communicable diseases (NCD) and common mental health conditions like depression (16, 17). The implementation support involved a technical assistance component, referred to as “Remote Coaching Support”, wherein an external coaching team would regularly meet with dedicated facility staff to enhance motivation, build capacity, and foster problem-solving efforts specific to the screening, referral, and care delivery for depression treatment as part of routine NCD care.

Technical assistance (TA) has been widely used across diverse settings and is a core “capacity-building” strategy for improving implementation outcomes (18). TA aims to enhance the motivation and capacity of individuals or teams (e.g., health workers) intended to adopt and integrate the evidence-based intervention (e.g., screening for depression as part of collaborative care) into their practice settings (e.g., primary health care facility). Generally, TA relies on an external person or group, ideally with training or knowledge in implementation science or improvement methods, to provide hands-on, individualized support through in-person or remote-based coaching, consulting, modeling, facilitation, referral to resources, and/or professional development specific to the intervention (19). Similarly, a range of models and approaches are available to support the ongoing quality management of implementation strategies, like TA, in healthcare settings. One such are continuous quality improvement (CQI) methods, which promote iterative changes for improving implementation of the strategy by ensuring the individual(s) continuously asks questions like “How are we doing?” and “Can we do better?” (20). However, putting any strategy into practice is a dynamic and relational process that involves addressing different contexts and varying norms among stakeholders within a given organization or setting. It requires careful consideration of not just what, when, where, and why of a strategy, but also how it can be tailored to the specific context to achieve successful outcomes.

Moreover, given context-specific facilitators and barriers that could enhance or diminish the impact of technical assistance, or any given support strategy, it is important to reliably document the systematic processes used to develop, adapt, and test strategies to increase learnings for cross-cultural applications of what works (21–25).

At the time of the launch of the ESSENCE program, the Ayushman Bharat program was in its initial stages of rollout, therefore being an optimal time for the Implementation Trial to generate new and practical insights for health system officials and policymakers on best practices for sustainable uptake of Ayushman Bharat, specifically for integrating mental health into primary care services. As part of the trial design, the Remote Coaching Support would be compared to “routine implementation support,” informed by existing service delivery and implementation support processes of the NCD and district health program. The design of the ESSENCE Implementation Trial is published elsewhere (14). In line with recommendations and to promote shared learnings on the development and adaptation for implementing technical assistance, this article documents the systematic design of the Remote Coaching Support strategy tailored to the context of the Ayushman Bharat initiative within primary care in Madhya Pradesh, India.

## 2 Materials and methods

The design of the implementation support strategy was guided by the evidence-based system for innovation support (EBSIS) framework. EBSIS is a theory-driven logic model that aims at identifying, designing, and testing four core capacity-building components (tools, training, technical assistance, and quality assurance/quality improvement) and their interactions impacting implementation uptake (26). The development of the technical assistance component was informed by 1. A mapping exercise of local health facility structures and non-communicable diseases programming followed by 2. A literature review to identify evidenced implementation support strategies using technical assistance, coaching methods, continuous quality improvement, and/or remote-based communication. We present these steps and their outcomes in sequence, as part of the methods, and then present the resulting strategy and delivery protocol.

### 2.1 Conduct a mapping exercise of local health facility structures and non-communicable diseases programming to determine existing capacities, processes, and implementation strategies with which the technical assistance could align

In 2019, a system mapping exercise was conducted to inform the development of the implementation strategy, including the technical assistance component and other processes related to strengthening the integration of the collaborative depression care package and ensuring alignment with existing programs. The goal was to understand the delivery process, information management system, and any implementation support mechanisms for primary care facilities that were implementing a non-communicable diseases (NCDs) program as part of the government initiative Ayushman Bharat, in the Sehore District of Madhya Pradesh, India.

To support the continuum of comprehensive care, the Ayushman Bharat initiative promotes a reorganization of service delivery into three tiers: family/household and community level, comprehensive primary care at facility level, and first referral level care, such as for emergency or specialized care, typically at a district-level hospital (DH). This reorganization includes the gradual transitioning of existing primary health care centers (PHCs) and sub-health centers (SHCs) into Health and Wellness Centers (HWCs) (27). Services across these tiers may overlap due to catchment areas and resources. The HWC facilities are intended to offer comprehensive primary health care services, including for NCDs, mental health, and emergency services, and support a continuum of care that connects at the community, primary, and district (or specialized) levels. Our mapping included 13 facilities that were recently transformed into HWC facilities, with transitions ongoing. The mapping was limited to service delivery and continuum of care for the NCDs diabetes and hypertension (screening, diagnosis, treatment, referral, and follow-up) as these processes were deemed as most aligned with the delivery of the collaborative depression care package. Both quantitative and qualitative research methods were used for data collection, including quantitative enumeration and profiling, observation of facility operations, and qualitative interviewing with a range of service providers and district health officials.

We summarize the mapping into four key categories of findings: (1) Facility profiles for HWCs of Sehore District, (2) NCD Program Service Delivery, (3) Information systems flow for the NCDs program, and (4) Implementation support mechanisms for the NCDs program.

#### 2.1.1 Facility profile for HWCs of Sehore District

Across the 13 facilities, there are large variations in catchment population size, staffing levels, and patient loads for general outpatient services and NCD screening. Training and delivery in mental health services and pharmacological medications for depression are not currently provided across the facilities, despite guidelines suggesting otherwise. In all 13 HWCs, at least one Medical Officer (MO) is designated as a facility lead for a range of responsibilities, including care supervision, case management, managing specialists’ referrals and medication supply, providing pharmacological treatment, and overall assessing team performance. Across facilities, at least one auxiliary nurse midwife or staff nurse, sometimes referred to as mid-level health providers (MLHP) is available to support a range of health care responsibilities, including prevention and promotion, and pre-natal and post-natal care. Finally, at least one pharmacist is available at facilities and a specific data entry operator is available in five facilities.

#### 2.1.2 NCD program service delivery

Services for the program are intended to work within the three-tiered continuum of care (community, primary, specialized), and delivered in the community (e.g., household visits) and facility level. Community-based activities for the NCD program are delivered by Accredited Social Health Activists (ASHAs) and include awareness generation about NCD risks and promotion of healthy lifestyles, household surveys and NCD risk assessments, and referral, or mobilization, of high-risk individuals to receive confirmatory screening, diagnosis, and treatment at the facility level. Facility-level NCD program activities are primarily led by MHLPs, such as ANMs or staff nurses. Activities include formal diagnostic screening and treatment of NCDs under the supervision of an MO, including any necessary investigations or medication, as well as referral or ambulatory services for emergency cases at central community health centers and/or district hospital based on geographic location and emergency need. So far, only one facility has a dedicated space for NCD services, although district authorities plan to open separate NCD “cells” for each HWC for improving operations. Both levels of care include follow-up (e.g., household screening, HWC visit) to manage risk and ensure treatment continuation; however, patient nonattendance or “no-shows” for follow-up appointments have been a major barrier to care continuance.

#### 2.1.3 Information system for the NCDs program

The facilities use a recently established digitized Health Management Information System (HMIS). HMIS is an electronic health record portal for entering, monitoring, and syncing data within and across facilities. An NCD application, usable on a tablet, and web-based versions (e.g., web portals) are available to support various levels of staff in entering data. A secondary portal available to district-level health officials allows syncing of data across facilities to support program planning and evaluation. A District Monitoring and Evaluation Officer (M&E) synthesizes the reports and presents the data during monthly district-level review meetings. Common challenges with the digital system include internet connectivity issues, technical glitches in uploading and synchronizing data, inability to track data overtime, lack of human resources and outdated tablets provided for data collection. As such, the 13 HWCs continue to keep paper-based health logs for back-up and ensuring consistent data records.

#### 2.1.4 Implementation support mechanisms for NCDs program

Various methods are used to support the implementation of the NCD program across the HWCs, both remotely and in-person. Overall, the mechanisms intend to support timely reporting, improve data quality, meet objectives for performance indicators, and improve quality of care; however, most is *ad-hoc* with no standardized processes in place. In-person methods included monthly or needs-based supervision visits, and weekly and monthly review meetings held by facility and community teams, respectively, to support with addressing barriers, problem-solving, and encouraging peer-based learning. Remote methods included needs-based telephone calls for any high-level or urgent issues with data monitoring or entry, and e-mail is used irregularly for sharing reports, directives, and meeting schedules. WhatsApp messaging was the most widely used method across all facilities, staff and levels of care, mainly for sharing performance indicator updates, training schedules, and facilitating communication among teams. There is no structured protocol for using WhatsApp, but several WhatsApp groups were iteratively established for different levels of participants, such as for higher-level leadership across facility and district-level units, or specific to staff levels, such as for ASHAS or MOs.

### 2.2 Conduct a literature review to identify evidenced implementation support strategies using technical assistance, coaching methods, continuous quality improvement, and/or remote-based communication

Literature was searched in April 2020 to identify various technical assistance strategies, communication technologies, and other quality improvement methods related to healthcare program implementation. PubMed, SCOPUS, CINAHL, Proquest: Healthcare, and Google Scholar were searched to identify relevant peer-reviewed articles and “grey literature” (e.g., websites, reports). A combination of search terms and keywords were used including: Technical assistance; Technical support; Quality assurance; Quality improvement; Implementation; Program Implementation; Service Delivery; Program Delivery; Mobile Technology; Digital Technology; Mobile Health; M-health; E-health. Literature that did not identify or use an implementation support strategy, was related only to research methodology, or consisted of “calls to action” without proposing a strategy to support implementation was excluded.

Thirteen articles were included, 2 of which were recent systematic reviews of continuous quality improvement or implementation strategies in health or mental health care, 9 were evaluations or protocols related to implementing specific remote or digitally-based support strategies, and two related to introducing theoretical models or specific training materials for implementing quality improvement techniques (3, 20, 28–38).

The implementation and quality improvement strategies identified in the literature were used for improving services related to either communicable or non-communicable diseases, including mental health and integrating depression interventions into primary care. In summary, the evidence from the 13 articles suggested that the benefits of continuous quality improvement methods and support strategies, such as technical assistance, for implementation outcomes are uncertain due to poor quality evaluations, complexities of healthcare systems, heterogeneous terminology related to strategies, and lack of detail on the application or specific steps to implement or monitor the strategies. Studies mentioned using an external coach, consultant, technical assistance team, or internal or external supervisors for technical assistance but were not specific in terms of how people were selected, trained or how much they cost. Authors of one of the systematic reviews (20) pointed out that the most effective approaches for impacting both clinical processes and patient outcomes involved a regular meeting schedule (e.g., weekly) with a designated team, engaging leaders in regular meetings, and using the Model of Improvement and the Plan-Do-Study-Act (PDSA) cycle to guide meetings, enhance communication and establish and evaluate goals for desired and continuous improvement. The Model of Improvement (38) has been previously evidenced as successful in a range of organizations in diverse settings, and comprises two parts: (1) Addressing three fundamental questions (What are we trying to accomplish?, How will we know that change is an improvement?, What change can we make that will result in improvement?) and (2) Using the PDSA cycle to test changes in real-world settings (28, 39, 40).

In terms of remote or digital methods of delivery, conventional SMS text messaging or free mobile application messaging (e.g., WhatsApp, Facebook Group, WeChat) were used for correspondence, as well as teleconferencing or video-based calls for meetings. Studies that used conventional SMS messaging, or in a few cases a digital platform, primarily used it for one-way correspondence to send reminders or “push” notifications for education, medical adherence, and counseling. Studies that used free mobile application messaging (e.g., WhatsApp) found it to be preferable to one-way messaging platforms, as it allowed for more interactive communication for the coaching and implementing teams, was easier to share information, like links, discussions, or photos, within the group, and seemed most accessible and appropriate in lower-resourced settings. Although WhatsApp was the most popular form of communication, studies highlighted that it should be used in tandem with teleconferencing or in-person meetings to establish relationships, generate ideas, build teamwork and communication patterns, and re-energize motivation and confidence among the implementers, especially when faced with barriers.

### 2.3 Development setting

The development of the implementation support strategy was conducted through a collaborative effort led by Sangath Bhopal Hub, in Bhopal, Madhya Pradesh, India, in partnership with Harvard Medical School, in Boston, MA, United States and implementation science experts from the University of North Carolina, Chapel Hill, NC, United States and Cincinnati Children’s Hospital Medical Center, Cincinnati, OH, United States. Sangath is a nonprofit organization focused on mental health research and implementation science and has undertaken significant work in strengthening mental health systems. The hub in Bhopal started operations in 2011 with a memorandum of understanding with the Directorate of Health Services, Government of Madhya Pradesh, for the PRIME project, and continues to extend the work in Sehore District and the rest of the state under ESSENCE and other projects.

## 3 Results

Building upon the evidence and key lessons learned from the PRIME study, the mapping and the literature review, we first summarize the main findings from the mapping exercise and literature review and then present the resulting contextually relevant technical assistance strategy and delivery protocol.

### 3.1 Summary findings from mapping exercise and literature review

The mapping exercise identified that NCD programming is not consistent across facilities, including varying numbers of dedicated staff and a lack of dedicated space. Existing implementation support is inadequately structured and primarily used on a needs basis, which may explain why there is sub-optimal performance of NCD indicators, such as follow-up care rates. Potential personnel were identified for screening and referral of depression cases (ANMs or staff nurses), for formal diagnosis, initiation of treatment, and referral (MOs) to community-based delivery of a psychosocial intervention (ASHAs). Technical assistance strategies therefore could support ANMs, staff nurses, and MOs in integrating depression care responsibilities into the NCD programming. Data collection and screening could align with the current HMIS platform and paper-based logs and support could be provided both in-person or remotely.

The literature review identified that the most effective implementation support strategies used regular meetings, guided by a continuous quality improvement method, primarily the Model of Improvement and Plan-Do-Act-Study (PDSA) cycle, which was enhanced by hired external coaches or consultants who could also motivate, build team communication, implementation skills, and problem-solve through both in-person and remote methods, including WhatsApp.

### 3.2 Design of the technical assistance support strategy

A summary of the resulting technical assistance strategy according to reporting criteria (*Name it; Define it; Operationalize it*) by Proctor and colleagues (25) can be found in Table 1. The technical assistance strategy was identified as “Remote Coaching Support.” Given the active use of WhatsApp in the health facilities and the evidenced use of remote methods and WhatsApp messaging from the literature review, the strategy would be delivered primarily through remote methods using coaching techniques, thereby aptly represented by the name Remote Coaching Support. A “strategy development team” was established for developing the protocol. The development team (*n* = 5) consisted of four individuals who had a minimum of master’s level training in public health and one senior international expert in applying tools and methods of implementation and improvement science to strengthen public health programs and health service delivery processes. Three of the five had experience with local systems in the Sehore District, of which one had more than 5 years of expertise in applying and teaching implementation science methods globally.

**Table 1:** Specification of the technical assistance strategy, Remote Coaching Support, according to Proctor et al. (25) criteria.

Domain	Strategy: remote coaching support (technical assistance)
*Name it*	Remote Coaching Support
*Define it*	Use technical assistance via externally hired and trained coaches to increase the motivation, skills, and performance of identified health facility staff (“health facility teams”) in the screening and referral of depression cases. Embed the strategy within ongoing facility processes (e.g., meeting structure) and use remote techniques, like WhatsApp, to facilitate integration within ongoing non-communicable diseases programming.
Operationalize it	
a) *The actor*	The target of the coaching (the actor) is determined as “facility teams” at primary care level, made up of health facility staff (auxiliary nurse midwifes [ANMs], staff nurses, and medical officers) that have the most regularly occurring non-communicable disease (NCD) indicator review meetings and are responsible for the screening, referral and treatment of depression within the NCD program as part of integrating the stepped collaborative depression care into primary care.
b) *The action*	An adapted Model for Improvement and the Plan-Do-Act-Study (PDSA) cycle will guide a systematic and iterative process for change, involving trained coaches to: 1. schedule regular meetings (coaching sessions) with facility teams that are in line with existing facility meetings, 2. prepare and plan for each meeting by reviewing facility data, setting performance goals, and preparing the coaching session agendas, 3. conduct coaching sessions to discuss data, facility team activities from previous week(s), review the PDSA cycle worksheet, and decide next steps to support facility teams in increasing regular screening and referral of patients; 4. fill coaching self-assessment (fidelity checklist) for continuous improvement of the Remote Coaching Support strategy.
c) *Action target*	Increase knowledge, skills and attitudes of health facility teams (ANMs, staff nurses, medical officers) in the screening and referral of depression cases during their NCD screening to increase the proportion of incoming patients that are screened, and of those screened positive, to increase proportion of cases referred for consultation and treatment as part of strengthening the integration of the stepped collaborative care model for depression into primary care.
d) *Temporality*	Remote Coaching Support will occur during real-world delivery of the collaborative depression care model within the ongoing NCD program at primary health facilities.
e) *Dose*	Remote Coaching Support sessions will occur bi-weekly for 30–45 min over Zoom or phone call, depending on preference and/or internet connection. Coaches will use pre-established WhatsApp messaging groups to communicate with facility teams for meeting times and sending reminders for preparing for the coaching session (e.g., review performance data, have your PDSA worksheet ready). Coaches and facility team members can flexibly use WhatsApp on a needs-basis for questions or recommendations during the 2-week cycle. Finally, coaches will host virtual “peer learning conferences” on a quarterly basis to facilitate cross-facility learnings and for collaboratively enhancing processes.
f) *Implementation outcome affected*	Uptake and fidelity of the screening and referral processes among facility teams within the collaborative depression care model as part of NCD program processes in primary health care facilities.
g) *Justification*	Evidence and key lessons learned from the PRIME study, the mapping and the literature review, including theoretically informed research, indicates that remote methods for delivering coaching as part of a technical assistance strategy that follows evidenced tools like the PDSA cycle, can enhance knowledge, skills and attitudes of health facility teams and lead to increased integration of the collaborative depression care model via increased rates of regular screening and referral for treatment of depression at primary care within the existing NCD program.

The strategy was designed in line with the EBSIS model, wherein external coaches would be trained to offer individualized support to facilities remotely (e.g., regular videoconferencing calls and WhatsApp messaging), guided by an adapted version of the Model for Improvement (MoI) that centers on implementation science theory, named “Model for Implementation” (41), and the PDSA cycles. Multiple HWCs were engaged for their interest in the trial; after which, 14 facilities were selected and randomized, 7 of which would receive Remote Coaching Support (14). The Remote Coaching Support would work with facility “teams” made up of staff that had the most regularly occurring NCD indicator review meetings (ANMs, staff nurses) and medical officers (MOs) given their leadership role. All team members would be asked to attend the calls, with a minimum of at least one team member per meeting, recognizing that these dedicated staff may have challenges in regular attendance due to ongoing duties in and outside of the clinic. Overall, the goal of the coaching is intended to increase the motivation, skills, and performance of facility team members for screening and referring cases of depression within their existing NCD program processes.

**Figure 1:**
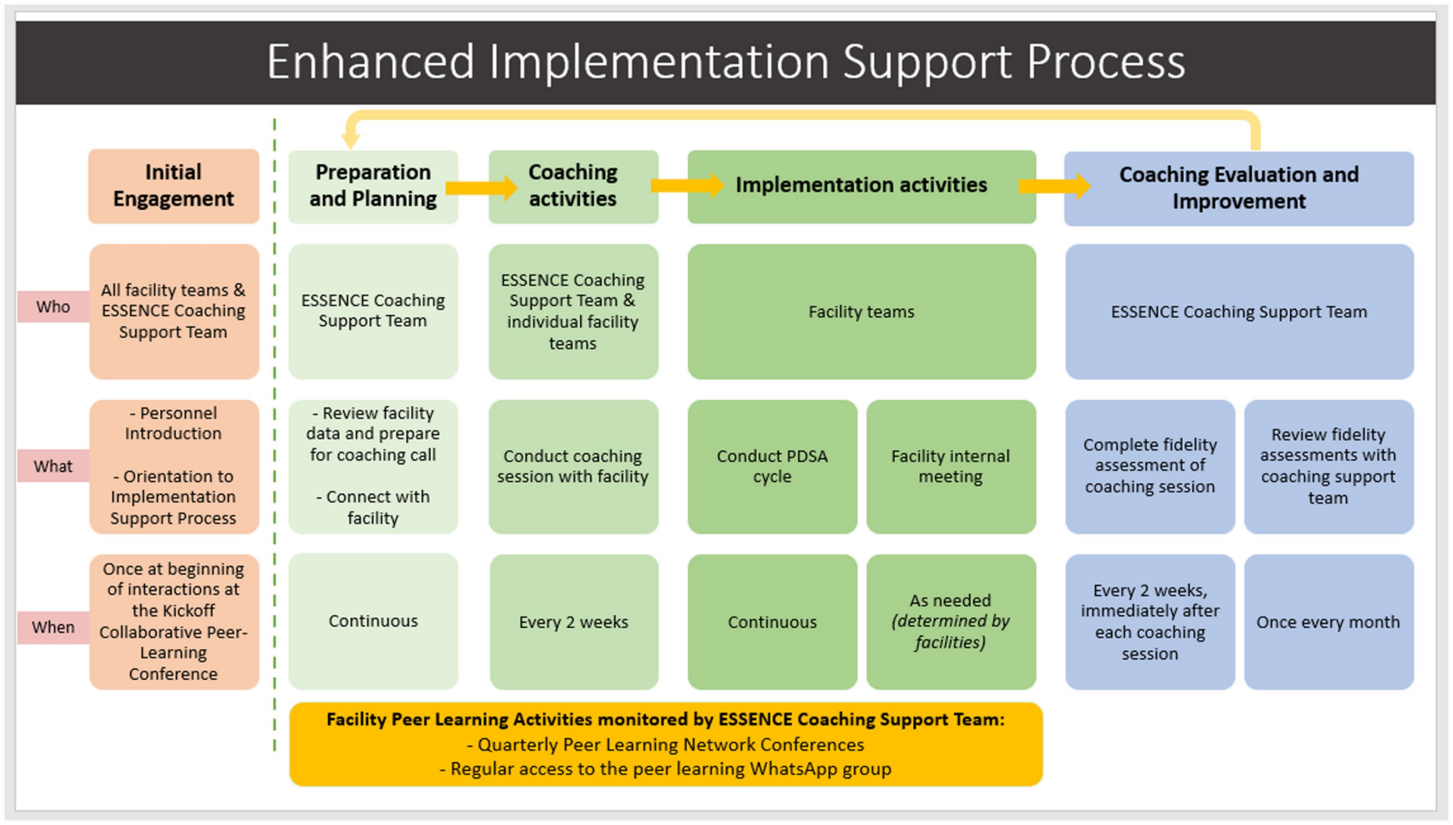
Diagram of the proposed “Remote Coaching Support” process.

A systematic framework for the proposed remote coaching support and “lifecycle” (see Figure 1) was designed using recommendations from the literature (24). An initial in-person engagement meeting was planned to establish relationships and orient the facility team to the specifics of the coaching support processes and overall implementation goals. Thereafter, coaching sessions between the coach and facility team were planned to occur bi-weekly for 30–45 min over Zoom or phone call depending on preference and/or internet connection. The sessions would involve establishing quality improvement goals, planning activities according to the PDSA cycle using a guided worksheet, discussing progress on activities, reviewing the PDSA worksheet, and planning steps for the next cycle. In between coaching calls, the coaches would review available facility data or background information and consider potential goals for the upcoming PDSA cycle. Additionally, they would use WhatsApp, with pre-established messaging groups, to find suitable times and confirm availability for the next meeting and send reminders for how to prepare for the coaching session (e.g., review performance data, prepare a goal, have your PDSA worksheet ready). Coaches could also use WhatsApp to follow up as needed with the teams for any questions or recommendations needed during the 2-week cycle. Finally, coaches would host virtual “peer learning conferences” every quarter to facilitate cross-facility learning and for collaboratively enhancing processes.

The strategy development team developed a reference manual (*see online appendix*) and tools for training remote support coaches. One expert from the development team led the training. A “coach delivery team” was established to support a cascaded training model and ensure sustainable coaching coverage. The coach delivery team (*n* = 3) included one “lead” coach with training in public health and experience working with local public health systems, and two “support” coaches with knowledge of local health systems and experience in mental health and implementation science research. All coaches were employed by Sangath. The lead coach would primarily be responsible for delivering the coaching and delivering training to new coaches, while the support coaches would assist the lead coach by taking notes during the facility team coaching calls, aggregating and reviewing facility data, and acting as “back-up” coaching coverage for the lead coach, as needed.

The reference manual for training the coaches resulted in a 20-page document covering a range of topics including basics of implementation and implementation support strategies, an introduction to the Model for Implementation, PDSA cycles and worksheets, purpose of coaches and their specific tasks, such as how to use PDSA worksheets in coaching meetings and using a fidelity checklist to promote self-reflection and improve coaching performance, structure of the proposed “Remote Coaching Support” process, and a module that covers tips on how to be a good coach, including specific leadership techniques for supporting behavior change. The final reference manual for training coaches can be found in the online Supplementary material.

**Figure 2:**
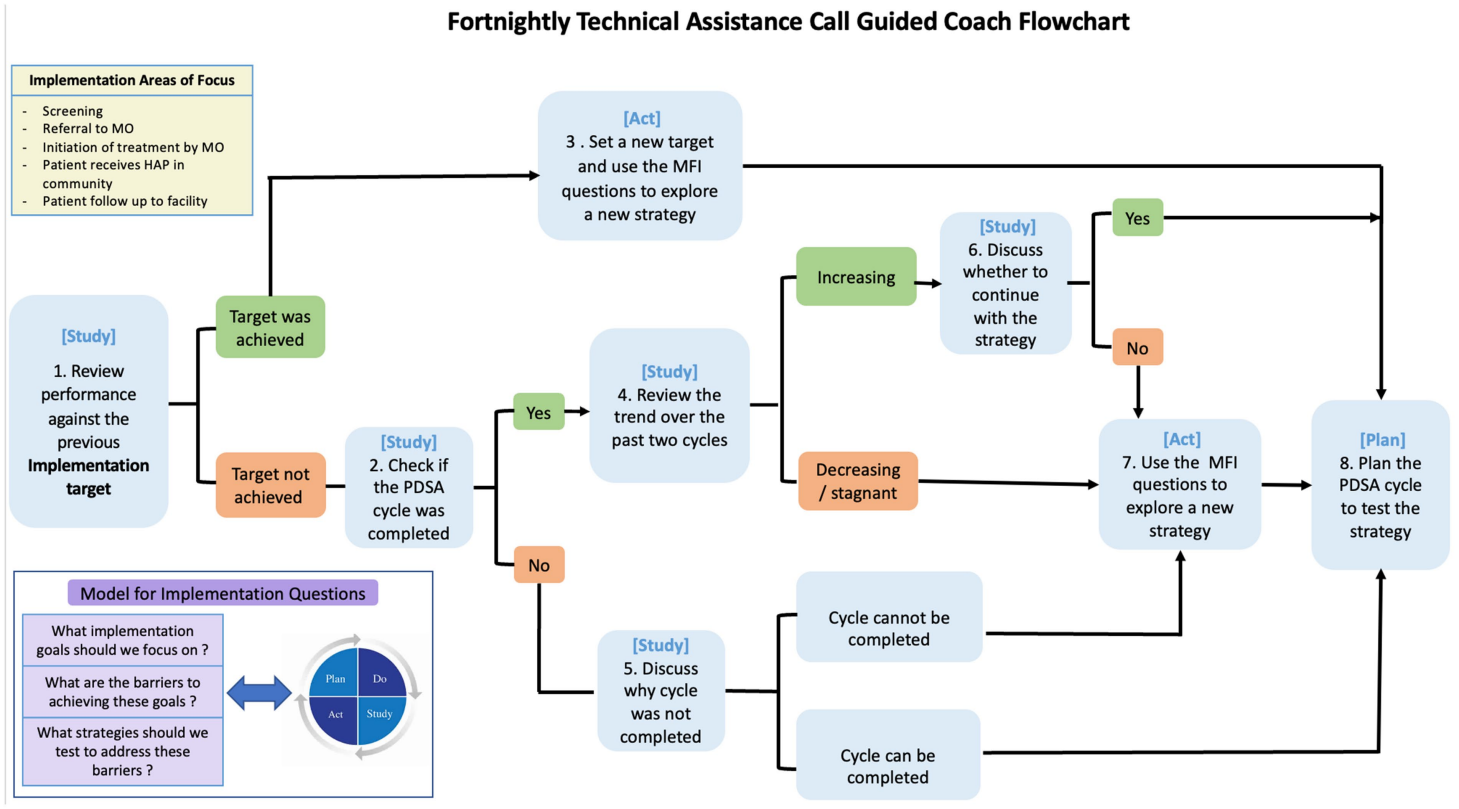
Decision flow chart to guide coaches in offering support during fortnightly calls.

Following a train-the-trainer model, an expert trainer from the strategy development team trained one person who was involved in the mapping exercise in a two-day remote training to become the “lead” coach. The training was guided by the reference manual and consisted of didactic sessions and roleplay exercises to practice coaching scenarios based on a suggested call schedule and decision flowchart for the coach (see Figure 2). Based on their experience with the local health systems, the trainee provided feedback and suggested minor changes for the coaching support process, including lessening the number of items on the PDSA worksheet and adding an item on the coach fidelity checklist to reflect on facilitators and barriers to coaching. After finalizing the materials, the newly trained lead coach used the materials to deliver a two-day in person training to two other people to become “support” coaches. Modeling an apprenticeship training model, a coaching supervision plan was established, wherein all coaches could meet every week with the expert trainers to discuss progress guided by subjective feedback and the coaching fidelity self-assessment. The supervision was planned to cover the first four PDSA cycles of the coaching meetings, with a tapering off determined by fidelity checklist scores and meeting topics.

## 4 Discussion

Contextually responsive implementation support strategies are needed to enhance the integration of mental health care in primary care facilities (3, 4, 9, 10). Technical assistance (TA) is a widely used capacity-building support strategy for strengthening the effective implementation of evidence-based interventions, like collaborative depression care, in routine health services; however, there is a lack of standards for systematically implementing and reporting technical assistance strategies globally (21–24). We present a description of a systematic process for developing an evidence-and-context-informed remote technical assistance strategy, identified as “Remote Coaching Support,” that will be applied to enhance the integration of a collaborative depression care package into primary care settings in alignment with a government non-communicable diseases (NCDs) care program in a district in rural India. Overall, the Remote Coaching Support aims to increase the motivation, skills, and performance of dedicated health facility staff for the screening and stepped care management of depression within their existing NCD processes, with the long-term goal of integrating comprehensive depression care (screening, referral, diagnoses, treatment) into routine NCD processes.

A core strength of our Remote Coaching Support approach lies in its adaptive and tailored design, developed through direct engagement with primary care providers and contextual assessments within the target settings. Our efforts involved mapping local health facilities, including brief interviews and discussions with dedicated staff, and conducting a literature review to gather insights into existing skill levels, challenges, and facilitators shared across facilities and those unique to each facility. This groundwork allowed us to tailor evidence-based practices into a technical assistance strategy that is responsive to varying skills and facility needs while aligning with the government’s newly minted Ayushman Bharat-Health and Wellness Centers programming. Additionally, to support opportunities for scale-up, we developed a reference manual and resources using a train-the-trainer and apprenticeship-informed model for training and supervising new coaches in delivering protocolized support (42, 43). To ensure reporting specification we followed Proctor and colleagues (25) recommendations for how we named, defined, and operationalized this strategy.

To effectively enhance the integration of mental health care in primary care settings, it is essential to recognize the diverse contexts in which strategies like Remote Coaching Support could be adapted and implemented, including varying levels of technical access and internet connectivity. According to best practices with quality improvement methods, the Remote Coaching Support protocol will be pragmatically rehearsed, wherein the coaching can be offered in “ramps” of increasing scale in real-world settings to support iterative adjustments and staged learning among the actor (health facility teams) (39, 44). For instance, in cases where remote modalities may not always be feasible or preferred, leveraging in-person approaches or using hybrid models that combine remote and face-to-face support could be considered.

Similarly, addressing the influence of policy and community-level factors, such as government support, funding allocation, and acceptability among local communities, is crucial for the success of implementation strategies aimed at integrating mental health interventions into primary care settings. In this study, we document our long-term commitment to buy-in, shared decision-making, and rigorous research that engages the voices of both government and community-based stakeholders to ensure the conduct of our research and design for strategies are in alignment with overarching values, policies, and planning, such as the governmental Ayushman Bharat initiative and needs of the primary health care workers we interviewed for this study.

Furthermore, the Remote Coaching Support will be implemented and evaluated as part of the ESSENCE Implementation randomized controlled trial, and the quantitative and qualitative results of its costs, effectiveness and acceptability will be published in a separate article. These future results will continue to contribute to further shared learning on the effectiveness and feasibility of this strategy and can act as a guide for other stakeholders or other implementation scientists.

## 5 Limitations

The development process enabled us to make iterative improvements through structured team meetings and feedback from practice trainings; however, there are a few limitations to the design and development process of the Remote Coaching Support. Given the lack of systematic implementation and reporting of technical assistance strategies and their components, such as dose, related specifically to integrating depression treatment into primary care, the design is heavily informed by theoretically driven models combined with practice-based knowledge, of which effectiveness depends highly on the responsiveness to the context. For instance, we use the Plan-Do-Act-Study (PDSA) cycle as a core component for the coaches to structure the meetings and motivational goals, given its flexibility and adaptability to real-world challenges in other healthcare strengthening initiatives (33, 39); however, the PDSA cycle does not guarantee users will achieve their desired outcomes (44). Although we plan to embed this cycle to strengthen the existing needs-based implementation support methods in the NCD program (e.g., WhatsApp messaging, regular meetings), introducing new techniques into an inadequately structured system may only result in key learnings for how quality improvement methods could work in these settings. Moreover, quality improvement methods have been cited to heavily rely on strong leadership and can be easily impeded in resource-constrained settings, like the facilities in the rural Sehore District (44–46).

## 6 Conclusion

In summary, this study outlines a structured, evidence-informed approach to developing the Remote Coaching Support strategy to enhance collaborative mental health care integration within primary care settings in the resource-constrained area of Madhya Pradesh, India. By emphasizing both consistency in implementation standards and adaptability to local contexts, this strategy seeks to foster scalable, context-responsive technical assistance that aligns with local health needs and government programming.

As we advance our collaborative care model and evaluate its effectiveness through the randomized controlled trial, we aim to provide detailed insights into the components and operational specifics to further clarify applications and outcomes for practitioners and stakeholders. Our approach underscores the importance of balancing systematic design with localized adaptation to accommodate the diverse regional contexts in which implementation may occur. We propose that future implementers take a similar approach, incorporating stakeholder engagement, documenting the selection and tailoring of strategies, and evaluating outcomes to support adaptation across diverse settings. Additionally, further studies could explore the scalability of this approach, documenting necessary adaptations and broader acceptability to extend the model’s reach across different health systems.

## Data Availability

The raw data supporting the conclusions of this article will be made available by the authors, without undue reservation.
