# Supplementary material for: Development of a remote implementation support strategy to enhance integration of depression treatment into primary care settings in rural India
Source: Front Public Health. 2024 Dec 6;12:1439997. doi: 10.3389/fpubh.2024.1439997 (PMC11659150; doi:10.3389/fpubh.2024.1439997)
Supplement: Supplementary file 1 [file Supplementary_file_1.docx]

**SUPPLEMENT 1.** ESSENCE Quality Improvement Reference Manual for training coaches in delivery of remote coaching support


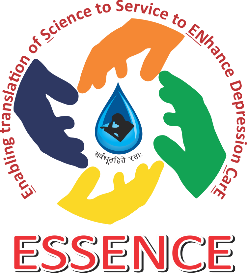


Quality Improvement Implementation Package for Integration of Mental Health Care Service Delivery

Reference Manual for Implementation Support Coaching Team

Written by implementation development team (Rohit Ramaswamy, Juliana Restivo, Vorapat Vorapanya, Shivangi Choubey, and Ameya Bondre)

Table of Contents

[1. Implementation Basics 4](#_Toc81404006)

[What is Implementation? 4](#_Toc81404007)

[What is needed to create a Successful Implementation? 4](#_Toc81404008)

[What is needed for Effective Implementation? 4](#_Toc81404009)

[2. Providing Implementation Support 5](#_Toc81404010)

[Model for Improvement 5](#_Toc81404011)

[7 STEPS to Develop the Model of Improvement 5](#_Toc81404012)

[Model for Implementation 6](#_Toc81404013)

[Defining Implementation Goals and Outcomes 7](#_Toc81404014)

[What are common barriers to achieving implementation outcomes? 7](#_Toc81404015)

[Common strategies to address barriers 8](#_Toc81404016)

[Next: testing strategies 8](#_Toc81404017)

[PDSA Worksheet Instructions: 9](#_Toc81404018)

[PDSA Worksheet Template: 10](#_Toc81404019)

[PDSA Worksheet Sample: 11](#_Toc81404020)

[3. Technical Assistance Coaching Tasks 12](#_Toc81404021)

[Technical Assistance Background 12](#_Toc81404022)

[Technical assistance coaching calls structure 13](#_Toc81404023)

[Initial engagement 13](#_Toc81404024)

[Preparation and planning for the technical assistance coaching calls call: 13](#_Toc81404025)

[Conducting the coaching session 14](#_Toc81404026)

[Following up after the Call 15](#_Toc81404027)

[Coaching Evaluation and Improvement 15](#_Toc81404028)

[4. Learning Collaborative Coaching Call 18](#_Toc81404029)

[5. Role of a Coach 19](#_Toc81404030)

[How to be a ‘good’ coach? 19](#_Toc81404031)

[Leadership Support Techniques for Coaches 21](#_Toc81404032)

[Dealing with coaching challenges 22](#_Toc81404033)

[References 23](#_Toc81404034)

# Implementation Basics

## What is Implementation?

- Implementation is “a specified set of activities designed to put into practice an activity or program” (Fixen, Naoom, Blase, Friedman, & Wallace, 2005).
- Implementation involves change to existing systems, procedures and processes to accommodate the new program or activity.

## What is needed to create a Successful Implementation?

According to Fixen, et. al., 2005 implementation appears most successful when:

- Carefully selected practitioners receive appropriate training, coaching and frequent and performance assessments.
- Organizations provide the infrastructure necessary for timely training, supervision and coaching, as well as, regular process and outcome evaluations.
- Communities are actively involved in the selection and evaluation of the program.
- Lastly, funding avenues, policies and regulations create a hospitable environment for implementation and program operations.
- In summary, to create the conditions necessary to implementation and sustain new practices and programs requires: decisions, actions and interventions.

## What is needed for Effective Implementation?

- Clearly defined infrastructure to **implement**
  - Available and staffed implementation teams
  - Defined roles and responsibilities
  - Motivation and capacity to implement
- Clearly defined infrastructure to **support** implementation
  - Data to identify implementation barriers
  - Implementation team to build the capacity to address implementation barriers
  - Coaching support for the implementation

What is Implementation Support?

Implementation support includes activities and processes to assist implementation teams in improving, scaling and sustaining evidence-based programs, practices and policies to improve outcomes. Implementation Support Teams should be assigned to provide Technical Assistance in a collaborative effort to select, tailor, and carry out contextually appropriate implementation strategies to enable implementation (Fleming, 2021). Technical Assistance (TA) is a professional development, coaching and mentoring, consultation, and other supports to programs and organizations to affect change or adoption of evidence-based or innovative practices (Dunst, 2019)

Responsibilities of Implementation Support Team according to Meyers, Durlak et. al., 2012

Include;

1. Increasing “buy-in” and motivation of implementation team
2. Building the implementation infrastructure and capacity
3. Assessing fidelity of implementation
4. Building linkages with external systems (e.g. district teams)
5. Facilitating problem-solving and sustainability of implementation teams

# Providing Implementation Support

## Model for Improvement


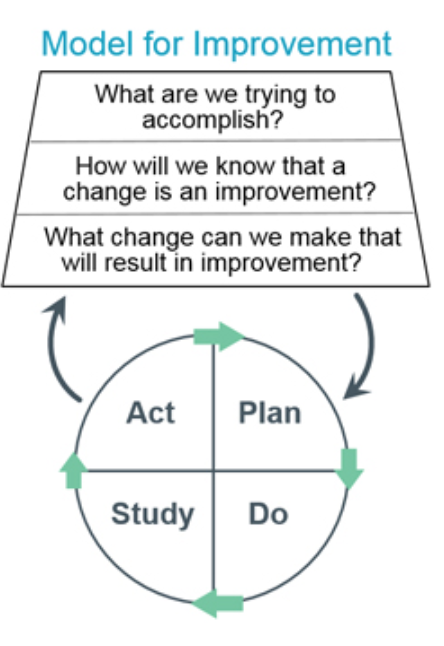
 The Model for Improvement (Langley et al., 2009) is a simple yet powerful tool for accelerating improvement. The model is meant to accelerate improvement but not meant to replace change models that organizations may already be using. This model has been used very successfully and universally by hundreds of health care organizations in many countries with diverse settings.(Taylor et al., 2014; Wagenaar et al., 2017) This model mainly aims to improve different health care processes and outcomes.

The model composed of two parts (as shown in Fig.1) :

- The first part includes following three fundamental questions. These three questions can be addressed in any order and team members should revisit anytime.
  1. What are we trying to accomplish?
  2. How will we know that a change is an improvement?
  3. What change can we make that will result in improvement?
- The Plan-Do-Study-Act (PDSA) cycle to test changes in real work settings. The PDSA cycle guides the test of a change to determine if the change is an improvement.

### 7 STEPS to Develop the Model of Improvement

**Figure 1** Model for Improvement

1. **Forming the Team:** Recruiting the right people on a process improvement team is critical to a successful improvement effort. Team must serve its own needs. [Each Facility should build its own team]
2. **Setting Aims:** The aim should be time-specific and measurable; it should also define the specific population of patients or other system that will be affected.
3. **Establishing Measures:** Teams use quantitative measures to determine if a specific change leads to an improvement. [Number of cases screened, referred, and treated]
4. **Selecting Changes:** Ideas for change may come from those who work in the facility or from the experience of others who have successfully improved.
5. **Testing Changes:** The Plan-Do-Study-Act (PDSA) cycle is shorthand for testing a change in the real work setting by (Langley et al., 2014) :

*PLAN:* Developing a plan to test the change

*DO:* Carrying out the test

*STUDY:* Observing and learning from the consequences

*ACT:* Determining what modifications should be made to the test

1. **Implementing Changes:** After testing a change on a small scale, learning from each test, and refining the change through several PDSA cycles, the team may implement the change on a broader scale — for example, for an entire pilot population or on an entire unit.
2. **Spreading Changes:** After successful implementation of a change or package of changes for a pilot population or an entire unit, the team can spread the changes to other parts of the organization or in other organizations. [Cross-facilities sharing]

## Model for Implementation

The Model for Implementation (Ramaswamy, 2019) provides a systematic approach to provide implementation support. The implementation support team should first answer these questions:

- 1. What implementation goals should we focus on?
  2. What are the barriers to achieving these goals?
  3. What strategies should we test to address these barriers?

When these questions are answered, the team can then proceed the implementation support process including:

- 1. Reviews implementation goals and looking at the data related to progress in achieving them
  2. Reinforces successful implementation activities
  3. Discusses on implementation gaps and challenges
  4. Facilitates approaches to address them


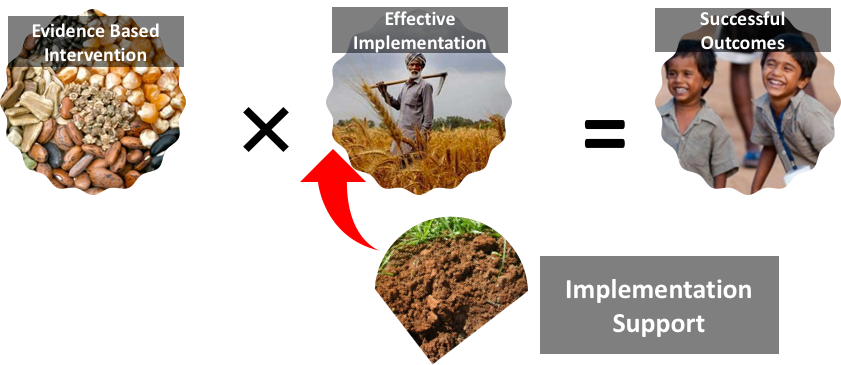


***Figure 2*** Diagram showing implementation support as a nutritious soils that plays a role in effective implantation which refer to as a farmer

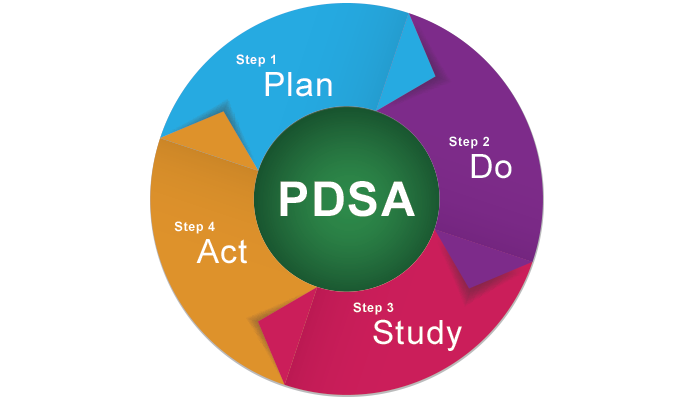


***Figure 3*** Model of Implementation (Ramaswamy, 2019)

## Defining Implementation Goals and Outcomes

- Implementation goals should be corresponded with expected Implementation outcomes. The Implementation outcomes are the results of implementation activities that measure the effectiveness of implementation.
- Implementation support team should be able to helps the teams identify implementation goals and measure appropriate implementation outcomes that the team desire.
- Example of Key Implementation Outcomes is shown in the **Table 1** below:

## What are common barriers to achieving implementation outcomes?

| **District barriers**   - Leadership Support - Staffing and Resources - Competing priorities | **Facility barriers**   - Leadership Support - Change tolerance - Resources - Competing priorities | **Provider barriers**   - Knowledge - Confidence - Motivation - Time | **Patient barriers**   - Knowledge - Beliefs and preconceptions - Access - Resources |
| --- | --- | --- | --- |

Adapted from Durlak and Dupree (2008)

## Common strategies to address barriers

## Next: testing strategies

1. To decide whether the proposed change will work in the actual environment of interest.

à ABC will work in the current situation of my/your facility

1. To decide which combinations of changes will have the desired effects on the important measures of quality.

à ABC with some amount of XYZ will increase screening by 20%.

PDSA Worksheet Instructions:
(Langley et al., 2009)

**Plan:** Plan the test, including a plan for collecting data.


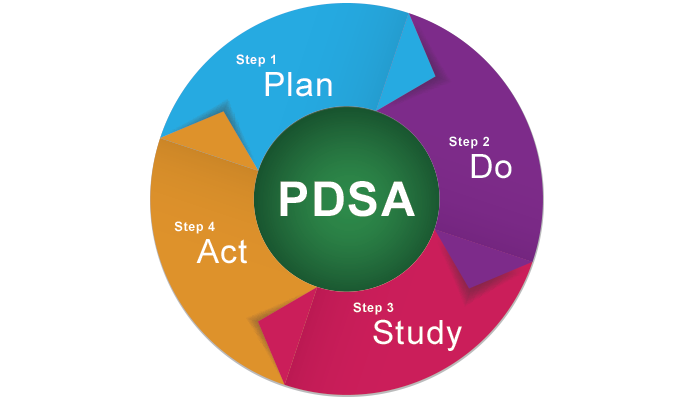


- - - - State the question you want to answer and make a
        prediction about what you think will happen.

**P**

- - - - Develop a plan to test the change. (Who? What? When? Where?)
      - Identify what data you will need to collect.

**Do:** Run the test on a small scale.


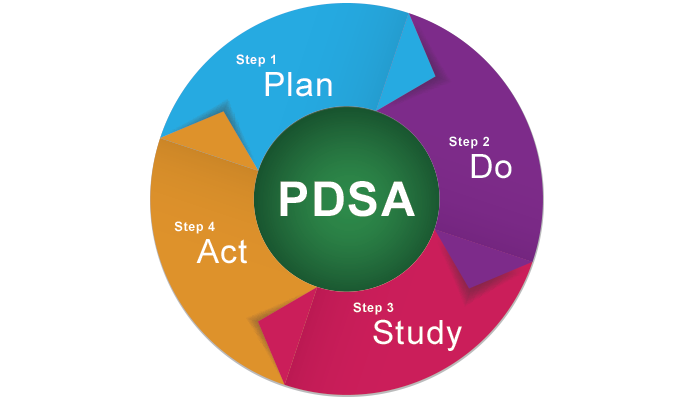


- - - - Carry out the test.
      - Document problems and unexpected observations.

**D**

- - - - Collect and begin to analyze the data.

**Study:** Analyze the results and compare them to your predictions.


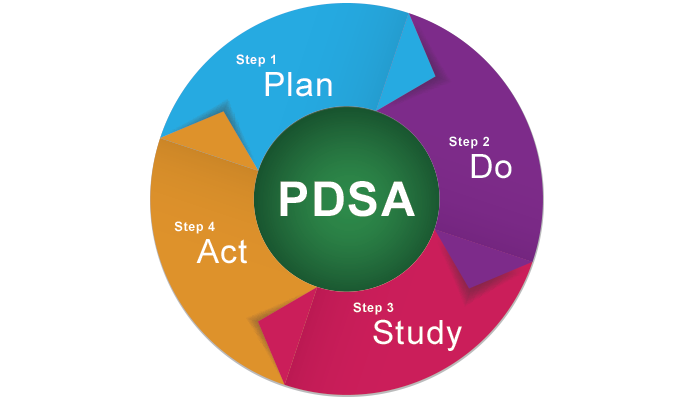


- - - - Complete, as a team, if possible, your analysis of the data.
      - Compare the data to your prediction.

**S**

- - - - Summarize and reflect on what you learned.

**Act:** Based on what you learned from the test, make a plan for your next step.


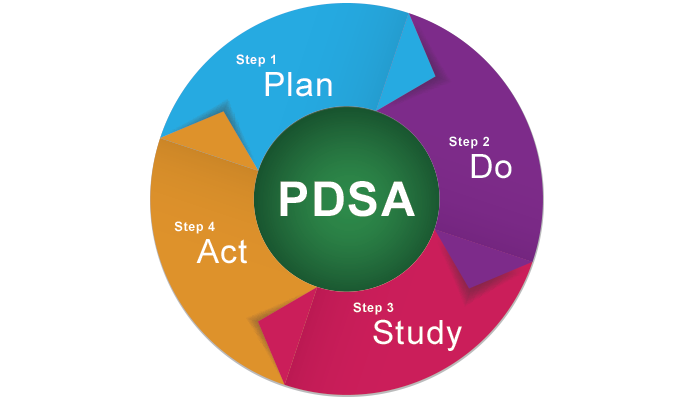


- - - - **Adapt** (make modifications and run another test), **adopt** (test the change on a larger scale), or **abandon** (don’t do another test on this change idea).

**A**

- - - - Prepare a plan for the next PDSA.

PDSA Worksheet Template:
(Langley et al., 2009)

| Cycle # | **What is the AIM Statement? What are we trying to test?** | | | | |
| --- | --- | --- | --- | --- | --- |
|  | **Objective**: | | | | |
| **PLAN:** Plan the test, including a plan for collecting data | | **List the tasks needed to execute this change** | **Responsible person** | **Date/Day to be performed** | **Place to be performed** |
|  |  |  |  |  |  |
|  |  | **Prediction:** | **Measures to determine if prediction succeeds** | | |
|  |  |  |  | | |
| **DO**:  Run the test on a small scale | | **Describe what actually happened. What did you observe?** | | | |
|  |  |  | | | |
| **STUDY:** Analyze the results and compare them | | **Describe the measured results and how they compared to the predictions. What did you learn? Did you meet your goal?** | | | |
|  |  |  | | | |
| **ACT:** Make a plan for your next step | | **What did you conclude? Describe what modifications to the plan will be made for the next cycle from what you learned. Determine what modifications you should make - Adapt, Adopt, Abandon** | | | |
|  |  |  | | | |

PDSA Worksheet Sample:
(Langley et al., 2009)

| Cycle 1 | **What is the AIM Statement? What are we trying to test?** | | | | |
| --- | --- | --- | --- | --- | --- |
|  | **Objective**: Test reducing the time spent on delivering the PHQ-2/PHQ-9 questionnaires to each patient, in hopes of increasing overall numberof patients screened. | | | | |
| **PLAN:** Plan the test, including a plan for collecting data | | **List the tasks needed to execute this change** | **Responsible person** | **Date/Day to be performed** | **Place to be performed** |
|  |  | 1. Holding the roleplay session and assessment 2. The constructive feedback provided at the end of the session by peer staffs. 3. **Plan for collecting data** :Each staff will time the PHQ-2/PHQ-9 screening session of the last patient on Wednesday -Friday and write a brief paragraph about their experience. | Staff (no’s 4) | Tuesday (afternoon) | Doctor/nurse Room |
|  |  | **Prediction:** | **Measures to determine if prediction succeeds** | | |
|  |  | Less time spent on the PHQ-2/PHQ-9, the more patients will be screened for depression. | - Comparing the time spent on PHQ-2/PHQ-9 - Analyzing the brief paragraph about their experience. | | |
| **DO**:  Run the test on a small scale | | **Describe what actually happened. What did you observe?** | | | |
|  |  | Four staffs were enrolled in the role play session on Tuesday. All of them found the session very helpful. They developed their own communication scripts of the PHQ-2/PHQ-9 screening questionnaire that were more consistent and appropriate. Devi and Priyanka were able to deliver the PHQ-2/PHQ-9 questionnaires within five minutes. Whilst, Usha delivered it on average of 8 minutes. Amita is a little bit over but she assures the team that she will keep practice and master her own lines. Number of patients screened was increased by 10% compared to last week. All of them felt more confidence in asking sensitive questions on PHQ-2/PHQ-9. | | | |
| **STUDY:** Analyze the results and compare them | | **Describe the measured results and how they compared to the predictions. What did you learn? Did you meet your goal?** | | | |
|  |  | **Prediction 1:** Less time spent on the PHQ-2/PHQ-9, the more patients will be screened for depression. *Result: each staff took 5-10 on average in delivering PHQ-2/PHQ-9 screening.*  **Prediction 2:** If we notice that a patient is unwilling to answer the question, we should rephrase the question and assure the patient that any information will be kept confidential. *Result: all of them felt more comfortable and confident in handling sensitive issues regarding the PHQ-2, PHQ-9.*  In addition to the team confirming both predictions, Amita has noticed that the screening table were in the middle of the clinic. She was sure that anyone could hear her questions and patient answers. She was afraid that her patients might not give an honest answer. | | | |
| **ACT:** Make a plan for your next step | | **What did you conclude? Describe what modifications to the plan will be made for the next cycle from what you learned. Determine what modifications you should make - Adapt, Adopt, Abandon** | | | |
|  |  | Everyone agreed to continue with this approach and keep the amount of time spending on PHQ-2/PHQ-9 within 5-10 minutes. The time limit for each session help increase the number of patients screened. | | | |

# Technical Assistance Coaching Tasks

## Technical Assistance Background

Technical assistance is associated with the level of supportive resources dedicated to implementation and operations including training and continuous supported coaching by trained program facilitators (Aschbrenner et al., 2019; Hodge & Turner, 2016). The goal of providing technical assistance is to enhance provider confidence in program delivery, build capacity, (Bustamante et al., 2012; Loman et al., 2010; Savaya & Spiro, 2012) and foster local problem-solving efforts (McDermott et al., 2003).

Technical assistance will include a 30-45-minute duration phone call with a quality improvement support coach and the facility team every two weeks.

*Figure 4* Diagram showing the process of Enhanced Implementation support including 1) Initial engagement 2) Implementation activities 3) Evaluation and Improvement

## Technical assistance coaching calls structure

*Figure 5* Diagram showing the standard technical assistance coaching calls structure

## Initial engagement

*Initial engagement between Coaching support team and facility teams:*

- Personnel introductions and relationship establishment
- Implementation support process orientation
- Occur once at the beginning of the support process

## Preparation and planning for the technical assistance coaching calls call:

*Contact with facilities:*

- Inform and remind each facility team about the date and time of the call via WhatsApp group
- Confirm the availability of the main point of contact and  additional team members if feasible.
- Remind the facility team to review their performance data, prepare a goal for the meeting, and be prepared to discuss their PDSA worksheet for the current cycle

*Data Review & TA Call Preparation:*

- Review in advance the performance data from each facility.
- Set a specific goal for the call and be prepared to suggest objectives to improve based on the performance data in case the facility doesn’t have any
- Follow the standard agenda for the call and leave space for the facility team to state their own goals for the call.

## Conducting the coaching session

*For the first coaching call:*

- Discuss with the team how to review the performance data and identify an improvement topic
- Brainstorm possible improvement topics that could be addressed in future PDSA cycles
- Revice the QI model for improvement and the PDSA worksheets (if needed)
- Introduce the structure for future calls

*Subsequent coaching calls:*

- Ask the facility team what their goals are for the call and what the key objectives that they would like to discuss about the past week
- Ask the facility team how the new program implementation is going and whether they have experienced any challenges during the integration process over the past week.
- Before reviewing your own goals first respond to any pressing issues raised or choose to add them to the agenda to discuss more formally.
- Share with the facility team your goal for the call and the agenda points you would like to cover
- Adhere to the agenda that you developed, but be flexible and adapt where necessary to be sure the facility team’s goals for the call are accomplished
- Review their PDSA worksheet for the current cycle and what they would like to work on for the next two weeks.
- If the team does *not* have their PDSA worksheet updated, review the performance data together and ask them to describe their progress and activities from the past two weeks.
- If the team is *not* able to identify a formal on-going improvement effort, review the performance data and brainstorm about barriers and improvement opportunities
- Remind the facility team to take both quantitative data (procedural indicators such as screening numbers, time spent with one patient etc.) and qualitative information (the team’s experience) into consideration as they discuss the issues for improvement
- Avoid directly steering the Facility team toward specific test change or solution.
- Focus the conversation on systems and processes during the session and avoid comments on individuals’ performance/competence unless the team mentions it.
- Show support and highlight the positive actions and successes that the team has made to motivate the facility to continue its work.
- Maintain a safe and comfortable environment for each of the facility personnel to be welcome to share their thoughts.
- Ask for feedback about how the coaching session can be improved.
- Remind the facility team about the next meeting

*Table 2* Agenda of the coaching call

*Figure 6* Technical Assistance Coaching Call Flowchart

## Following up after the Call

- Coaches can continue communication via the WhatsApp group with the facility teams between coaching calls to discuss how recommendations are implemented and if there are any questions.

## Coaching Evaluation and Improvement

*Self-assessment:*

- Fill out fidelity assessment immediately after *each* coaching session​
- Self-reflect from fidelity assessment
- Strategize how to improve the *next* coaching session or maintain the performance

*Review fidelity assessments with coaching support team:*

- Conduct review with peer coach
- Occur at least every two months

# Learning Collaborative Coaching Call

# Role of a Coach

The key roles of Quality Improvement Support Coach are facilitator, trainer, and Quality Improvement (QI) expert:

- **As a facilitator,** the coach keenly observes the team’s work and provides both positive and constructive feedback on team members interaction and work. The coach monitors the team through the Model for Improvement and intervenes appropriately to help the team move forward towards the desired objective. If necessary, the coach will work with each individual team member on the behavior changes necessary for the newly established work standards.
- **As a trainer,** the coach teaches the team the necessary technical and analytical skills to improve health care processes and outcomes. To better advise the team members, the coach should first explain the task or activity to be learned and the reasoning behind why it is important. The coach can then demonstrate the activity by using examples.
- **As a QI expert**, the coach provides guidance for understanding the QI principles or tools (e.g., teamwork, or preparing and analyzing a process diagram); identifying and documenting changes; and understanding and mastering data monitoring (both collection and analysis). (Integrated Family Health Program (PISAF) coaching manual. Coaching as a tool to support Quality improvement teams., 2011)

## How to be a ‘good’ coach?

***Do:***

**1. A good coach is self-aware.**To understand oneself, one’s coaching style, and how it is perceived and received by employees, is a critical first step to becoming a valuable and effective coach. Self-awareness is a journey unto itself, so we’ll be writing more about that in the coming weeks.

**2. A good coach brings specific and well-defined issues to the attention of others.**Being unspecific about problem areas, or failing to bring them up with the appropriate parties, suggests a reluctance to affect positive change and a lack of leadership.

**3. A good coach prepares for each session with information, examples, ideas, etc., and is ready for discussion.**
Coaching sessions should be scheduled in advance, and the coach should have a solid agenda for each session that lays out the mission for the day. Without structure, the coaching session can devolve into a casual conversation with no real substance or direction.

**4. A good coach treats individuals as partners in the organization, encouraging their input and trusting them to carry out assignments.**Some coaches are fans of “tough love,” while others are more lenient, but what all good coaches have in common is respect for their mentees. Contempt and resentment have no place in an effective coaching relationship, and only breed further conflict.

**5. A good coach knows the strengths and weaknesses of his or her employees.**Much like the coach of a sports team, he or she knows how to tap into the individual strengths of employees to get the most out of them and to get the greatest amount of productivity from the team, collectively and individually.

**6. A good coach makes expectations clear at the beginning of the coaching session.**Both the coach and the employee must have a sense that this meeting has a distinct purpose, and must agree on what that purpose is, for the session to proceed smoothly.

**7. A good coach allows enough time to adequately discuss issues and concerns.**Blocking out enough time for a solid session, rather than squeezing it in and rushing through, shows respect for the employee’s time and allows them to participate more thoughtfully.

**8. A good coach seeks out ideas and makes those ideas part of the solution.**Take it as a red flag if a coach is not willing to hear ideas, suggestions, or thoughts from other members of the team. A coach is there to serve the employees, not for the employees to serve his or her ego.

**9. A good coach listens to others and tries to understand their points of view.**Rather than assigning blame or delivering unhelpful criticism, he or she allows the employee to explain things from the other side, which can often uncover the root of a misunderstanding or miscommunication.

**10. A good coach expresses encouragement and optimism when both easy and difficult issues are discussed.**Sometimes an issue can be the elephant in the room that nobody wants to talk about. It’s the coach’s job to make this issue less intimidating by modeling a constructive attitude that brings the team together to address it.

**11. A good coach directly asks for a commitment to solutions that have been agreed upon.**Coaches can’t be wishy-washy about their expectations. If the employee isn’t held accountable for improving, it becomes a waste of everyone’s time to continue coaching.

**12. A good coach provides the resources, authority, training and support necessary for others to carry out solutions.**Coaching doesn’t end when the session ends. It is up to the coach to follow through with any additional guidance the employee might need to move forward.

**13. A good coach offers support and assistance to those he or she is coaching to help them implement change and achieve desired goals.**Professional development is a team effort. It’s usually not wise to simply cut the employee free after a session and expect him or her to achieve everything on their own.

**14. A good coach follows up on coaching sessions in a timely manner.**It’s all too easy for coaching to fall down the priority ladder among all the other demands of a manager’s day-to-day job duties. At the end of each coaching session, it’s a good idea to go ahead and schedule the next one, and to hold to that commitment when the time comes around.

**15. When solutions do not turn out as expected, a good coach proactively helps to define alternative actions.**If at first the employee does not succeed, it could be that there was a misunderstanding, or it could be that the original solution was a mismatch for that particular employee. A good coach is open to having a backup plan (or two).

***Do Not:***

1. Focus on the person, but on the system
2. Use judgmental language and embarrass the team
3. Provide solutions without input from the team
4. Create a plan without input from the facility teams
5. Talk more than you listen

## Leadership Support Techniques for Coaches

Transformational leadership is a style of leadership that focuses on empowering internal change within groups or organization (Burns, 1978). There are psychological mechanisms that underlie transformational and transactional leadership. Below are four elements of transformational leadership (Bass, 1985). If followers of a leader can feel trust, admiration, loyalty and respect for the leader because of these four elements, then the followers are more likely and willing to work harder than originally expected (Bass, 1985). These four elements include 1) Idealized Influence or Charisma; 2) Inspirational Motivation; 3) Intellectual Stimulation; 4) Individual (Personal) attention (Bass, 1985):

**1. Idealized influence (II) or Charisma:** the coaches serve as a role model for followers and embody the qualities they want in their followers. As a quality improvement coach helping to implement the integration of mental health care service delivery, coaches should have reliable knowledge of quality improvement activities and express the ability to deliver the needed assistance. Building trust between the coach and the facility team will help the coach influence the team to a certain degree. In this leadership style, the coach must be aware not to overinfluence or steer the facility team into certain direction.

**2. Inspirational motivation (IM):** the coaches have the ability to empower and inspire followers with simple words. Clarity is the key. By using this Quality Improvement Manual as a guide, the coaches should be able to deliver and provide guidance to the facility team according to its settings. Coaches may be required to customize the approach for different facilities depending on the needs of each facility. Combining the Idealized Influence (II) and Inspirational Motivation (IM) are what comprises the coaches’ charisma.

**3. Intellectual stimulation (IS):** as transformational leaders the coaches are responsible for challenging the facility team members to be innovative and creative by using PDSA cycles as a tool. The quality improvement support coach should challenge the facility team to higher levels of performance that align with meeting the goals to integrate the mental health care package effectively. This could include increasing the number of patients at the facility who are treated for depression which further increasing the number of individuals who ultimately receive treatment. The coaches can also encourage facility leaders to work with all members of the team and challenge them to provide feedback and ideas for improvement with the full staff. As the coaches challenge the facility leaders to think in this way it can increase the sustainability of the new integrated program for the long term (Hodge & Turner, 2016).

**4. Individual (Personal) attention (IA):** the coaches should demonstrate genuine concern for the needs, goals, and the concerns of each member of the facility team. This personal attention will help coaches and each of the team members develop interpersonal and professional trust. For example, the coach will feel more comfortable talking to the whole group and also have a one-on-one conversation with an individual member working at the facility if the wish. From this perspective, the coach can work towards training and assisting a member of the facility team who may be having difficulties or may need help with a specific task. When all staff and facility team members are involved in the integration of a new program it ensures a smooth implementation process and can lead to prolonged program sustainability (Hodge & Turner, 2016).

## Dealing with coaching challenges

1. **Manage your emotions**
   - Recognize your own preconceptions about people who are ‘challenging’
   - Keep a neutral mind
2. **Believe change is possible!**
3. **Take an alternative view**
   - Put yourself into their shoes

# References

Aschbrenner, K. A., Pratt, S. I., Bond, G. R., Zubkoff, L., Naslund, J. A., Jue, K., Williams, G., Kinney, A., Cohen, M. J., Godfrey, M. M., & Bartels, S. J. (2019). A virtual learning collaborative to implement health promotion in routine mental health settings: Protocol for a cluster randomized trial. *Contemporary Clinical Trials*, *84*. https://doi.org/10.1016/j.cct.2019.105816

Bass, B. (1985). *Leadership and Performance*. Free Press.

Burns, J. (1978). *Leadership* (1st ed.). Harper & Row.

Bustamante, R., Hurtado, E., & Zeribi, K. (2012). *Improving the Quality of Guatemala’s Public Health System: A View to Institutionalization*. *June*.

Hodge, L. M., & Turner, K. M. T. (2016). Sustained Implementation of Evidence-based Programs in Disadvantaged Communities: A Conceptual Framework of Supporting Factors. *American Journal of Community Psychology*, *58*(1–2), 192–210. https://doi.org/10.1002/ajcp.12082

*Integrated Family Health Program (PISAF) coaching manual. Coaching as a tool to support Quality improvement teams.* (2011). https://www.usaidassist.org/sites/assist/files/pisafcoachingmanual_english_2011_final

Langley, G., Moen, R., Nolan, K., Nolan, T., Norman, C., & Provost, L. (2014). *The Plan-Do-Study-Act ( PDSA ) Worksheet* (2nd ed.).

Langley, G., Nolan, K., Nolan, T., Norman, C., & Provost, L. (2009). *The Improvement Guide: A Practical Approach to Enhancing Organizational Performance* (2nd ed.). Jossey-Bass Publishers.

Loman, S. L., Rodriguez, B. J., & Horner, R. H. (2010). Sustainability of a Targeted Intervention Package: First Step to Success in Oregon. *Journal of Emotional and Behavioral Disorders*, *18*(3), 178–191. https://doi.org/10.1177/1063426610362899

McDermott, R., Tulip, F., Schmidt, B., & Sinha, A. (2003). Sustaining better diabetes care in remote indigenous Australian communities. In *British Medical Journal* (Vol. 327, Issue 7412, pp. 428–430). BMJ Publishing Group. https://doi.org/10.1136/bmj.327.7412.428

Peynetti Velázquez, P., Gupta, G., Gupte, G., Carson, N. J., & Venter, J. (2020). *Rapid Implementation of Telepsychiatry in a Safety-Net Health System During Covid-19 Using Lean*. https://doi.org/10.1056/CAT.20.0319

Savaya, R., & Spiro, S. E. (2012). Predictors of Sustainability of Social Programs. *American Journal of Evaluation*, *33*(1), 26–43. https://doi.org/10.1177/1098214011408066

Taylor, M. J., McNicholas, C., Nicolay, C., Darzi, A., Bell, D., & Reed, J. E. (2014). Systematic review of the application of the plan-do-study-act method to improve quality in healthcare. In *BMJ Quality and Safety* (Vol. 23, Issue 4, pp. 290–298). BMJ Publishing Group. https://doi.org/10.1136/bmjqs-2013-001862

Wagenaar, B. H., Hirschhorn, L. R., Henley, C., Gremu, A., Sindano, N., Chilengi, R., Hingora, A., Mboya, D., Exavery, A., Tani, K., Manzi, F., Pemba, S., Phillips, J., Kante, A. M., Ramsey, K., Baynes, C., Awoonor-Williams, J. K., Bawah, A., Nimako, B. A., … Pio, A. (2017). Data-driven quality improvement in low-and middle-income country health systems: Lessons from seven years of implementation experience across Mozambique, Rwanda, and Zambia. *BMC Health Services Research*, *17*(S3), 830. https://doi.org/10.1186/s12913-017-2661-x
